# Comprehensive Imaging Characterization of Colorectal Liver Metastases

**DOI:** 10.3389/fonc.2021.730854

**Published:** 2021-12-07

**Authors:** Drew Maclean, Maria Tsakok, Fergus Gleeson, David J. Breen, Robert Goldin, John Primrose, Adrian Harris, James Franklin

**Affiliations:** ^1^ Department of Radiology, University Hospital Southampton, Southampton, United Kingdom; ^2^ Department of Medical Imaging, Bournemouth University, Bournemouth, United Kingdom; ^3^ Department of Radiology, Oxford University Hospitals, Oxford, United Kingdom; ^4^ Department of Oncology, Oxford University, Oxford, United Kingdom; ^5^ Department of Metabolism, Digestion and Reproduction, Imperial College London, London, United Kingdom; ^6^ Department of Surgery, University Hospital Southampton, Southampton, United Kingdom; ^7^ Academic Unit of Cancer Sciences, University of Southampton, Southampton, United Kingdom

**Keywords:** colorectal (colon) cancer, liver, metastasis, radiomic biomarkers, MRI, computed tomography

## Abstract

Colorectal liver metastases (CRLM) have heterogenous histopathological and immunohistochemical phenotypes, which are associated with variable responses to treatment and outcomes. However, this information is usually only available after resection, and therefore of limited value in treatment planning. Improved techniques for *in vivo* disease assessment, which can characterise the variable tumour biology, would support further personalization of management strategies. Advanced imaging of CRLM including multiparametric MRI and functional imaging techniques have the potential to provide clinically-actionable phenotypic characterisation. This includes assessment of the tumour-liver interface, internal tumour components and treatment response. Advanced analysis techniques, including radiomics and machine learning now have a growing role in assessment of imaging, providing high-dimensional imaging feature extraction which can be linked to clinical relevant tumour phenotypes, such as a the Consensus Molecular Subtypes (CMS). In this review, we outline how imaging techniques could reproducibly characterize the histopathological features of CRLM, with several matched imaging and histology examples to illustrate these features, and discuss the oncological relevance of these features. Finally, we discuss the future challenges and opportunities of CRLM imaging, with a focus on the potential value of advanced analytics including radiomics and artificial intelligence, to help inform future research in this rapidly moving field.

## Introduction

Colorectal cancer (CRC) remains the second leading cause of cancer-related death in the developed world (1). Liver metastases are a major cause of death in patients with CRC and therefore optimising treatment of colorectal liver metastases (CRLM) is an important target for future research. Approximately 15% of patients will have synchronous liver metastases at initial diagnosis, with up to half developing liver metastases during their clinical course (2).

There are two main therapeutic strategies for CRLM. For those patients with polymetastatic disease, palliative systemic therapy is the mainstay of treatment. In patients with ‘oligometastatic’ disease (3), curative-intent surgical or image-guided treatment can be offered, often following neoadjuvant chemotherapy. Five-year survival rates following hepatic metastasectomy for CRLM are 28-49% (4), with some long-term survivors, in contrast to patients with polymetastatic disease who have a 1-year survival rate of approximately 55%, and 5-year survival rate of 3% (2). Historically, stricter surgical criteria have limited patient numbers proceeding to resection but, as evidence emerges that even patients with extensive disease derive benefit from local treatment (5), more patients are being offered treatment with curative intent. Selecting which patients who may benefit from radical treatment is an important challenge for clinicians, given that a significant proportion of patients undergoing metastasectomy suffer early relapse with incurable disease (4, 6). In the polymetastatic setting, multiple agents, including various cytotoxic regimens, targeted treatments and immunotherapies have transformed the options for patients without a curative option (7). Anticipating which agents will work optimally for each individual patient is critical, especially considering a counterintuitive response of patients to many of these therapies (8), which underlines the need for a more detailed assessment of colorectal metastases prior to commencing therapy.

The pathological literature describes the varied histopathological features of CRLM, both their internal architecture and their interface with the surrounding liver parenchyma (9, 10). Several histopathological and immunohistochemical phenotypes are associated with differential prognostic outcomes (10, 11). Unfortunately, histological phenotypic information is principally only available *after* resection, and is therefore of limited value in pretreatment prognostication, or when planning the neoadjuvant or polymetastatic treatment.

Improved techniques for *in vivo* disease assessment, which can characterise the variable tumour biology, would allow clinicians to plan personalized management strategies. Imaging already plays a central role in assessing the sites and burden of metastatic cancer both before and after treatment (12). Advanced imaging techniques, in particular multiparametric MRI (mpMRI) and functional imaging techniques, combined with novel image analysis techniques, have the potential to improve disease characterisation, and the advantage of being non-invasive, repeatable, and with the potential to assess all tumour sites.

In this narrative review, we outline how imaging techniques could reproducibly characterize the histopathological features of CRLM, with several matched imaging and histology examples to illustrate these features and discuss the oncological relevance of these features. We discuss the future opportunities and challenges of CRLM imaging, with a focus on the potential value of advanced analytics including radiomics and artificial intelligence, to help inform future research in this rapidly moving field. This review was informed by searching PubMed for relevant papers using search terms including ‘colorectal’, ‘liver metastas*’, ‘MRI’, ‘CT’, ‘PET’ and ‘imaging biomarker’ and a search of references.

## Imaging of CRLM

Contrast enhanced CT (ceCT) is the mainstay of oncological imaging, and is the first line test for staging, surveillance and response assessment. Where there is diagnostic uncertainty, or precision about the number of metastases is crucial, mpMRI is the gold-standard technique for detecting and characterising focal liver lesions. Standard sequences include unenhanced T1- and T2-weighted sequences (including opposed-phase imaging), diffusion weighted imaging (DWI) and multiphase acquisitions following intravenous extracellular gadolinium chelate contrast media or liver-specific contrast agents. For CRLM, multiparametric liver MRI has superior per patient and per lesion sensitivity to CT (13, 14), and provides higher per lesion sensitivity than ^18^F-fluorodeoxyglucose-positron emission tomography/CT (FDG-PET/CT) (13). Additionally, it provides high quality anatomical information which is invaluable for treatment-planning. It is therefore recommended as part of routine imaging work-up for patients being staged prior to liver-directed therapy (15). 18F-fluorodeoxyglucose-positron emission tomography/CT (FDG-PET/CT) is often used in patients with CRLM to detect extrahepatic disease that would preclude a radical treatment approach (16).

## Radiopathological Features of CRLM

### Tumour Interface With Normal Liver

Arguably the most clinically-relevant histopathological feature amenable to evaluation by imaging is the interface between normal liver and tumour. Interface features could influence chemotherapy selection, in particular for antiangiogenic agents (17, 18), inform surgical approach (19) and improve risk stratification for recurrence and overall survival (20, 21). These features are also well covered in another recent review (22).

**Figure 1:**
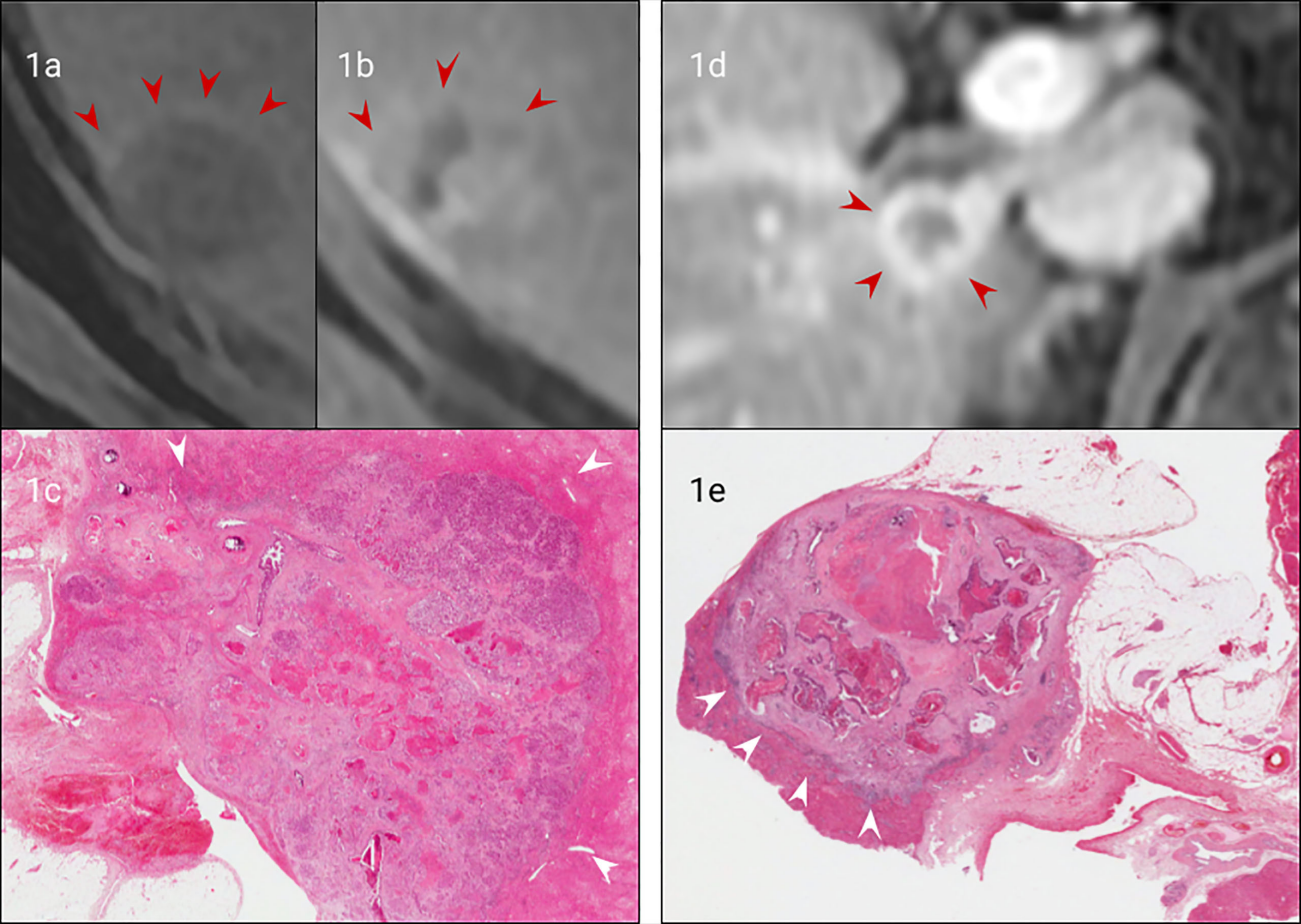
Matched imaging and histology of non-encapsulated **(A–C)** vs capsulated CRLM **(A, D, E)**. Arterial phase gadolinium-enhanced T1 fat saturated MRI of a CRLM with early peripheral enhancement indicating compression of hepatocytes (arrows), **(B)**. Portal venous gadolinium-enhanced T1 fat saturated MRI showing an absence of peripheral enhancement with isointensity to normal liver (arrows), **(C)**. H&E staining (from **(A, B)** confirming no true capsule with peripheral compression of hepatocytes (arrows). **(D)**. Portal venous phase gadolinium-enhanced T1 fat saturated MRI demonstrating clear peripheral enhancement of a fibrotic capsule (arrows), **(E)**. H&E staining of the CRLM (from **(D)** confirming a true fibrotic capsule/desmoplastic interface (arrows).

The growth pattern of CRLM has been subdivided into three patterns of interface with the liver parenchyma: ‘pushing’, ‘desmoplastic’ and ‘replacement’ (9). The ‘pushing’ pattern is characterized by direct abutment of tumour cells on the liver parenchyma, with expansile growth flattening the liver plates. The ‘desmoplastic’ interface (present in approximately 40%) is differentiated by a rim of desmoplastic, fibrotic stroma incorporating a lymphocytic infiltrate, numerous bile ducts and capillaries (**Figure 1E**). This classification includes the term ‘pseudo-encapsulated metastases’ and has been linked to improved outcomes compared with non-desmoplastic metastases (17, 18, 20, 21, 23). It has also been suggested a less aggressive surgical approach, with a narrower margin, can be attempted with desmoplastic lesions, thus sparing more normal liver (19). The ‘replacement’ growth pattern is characterised by intimate contact between tumour cells and hepatocytes with a preserved reticulin pattern within the tumour tissue. Growth of these lesions therefore appears to be *via* vascular co-option, rather than angiogenesis, which is supported by their proven poor response to anti-angiogenic agents (17, 18, 23).

There are several potential imaging correlates that could predict the presence of these patterns *in vivo.* The presence of a peripheral fibrotic capsule can be indicated by MRI (**Figure 1D**) (24–27), given typical MRI characteristics of fibrous tissue which is typically low T1 and T2 signal, and accumulates contrast on delayed contrast enhanced imaging (e.g. **Figure 1**). These features may reliably distinguish desmoplastic from other tumour types, although further research is required to establish the utility of this as a diagnostic tool.

Hepatocyte-specific contrast agents could also be useful in determining a ‘replacement’ growth pattern. This is similar to how microvascular invasion (MVI) can be identified with hepatocellular carcinoma (HCC) as reproducible hypoperfusion of liver parenchyma surrounding the tumour due to subtle tumour infiltration (28). However, this feature is currently untested in ‘replacement’ interface patterns of CRLM.

### Vascular, Biliary, Lymphatic and Perineural Invasion

**Figure 2:**
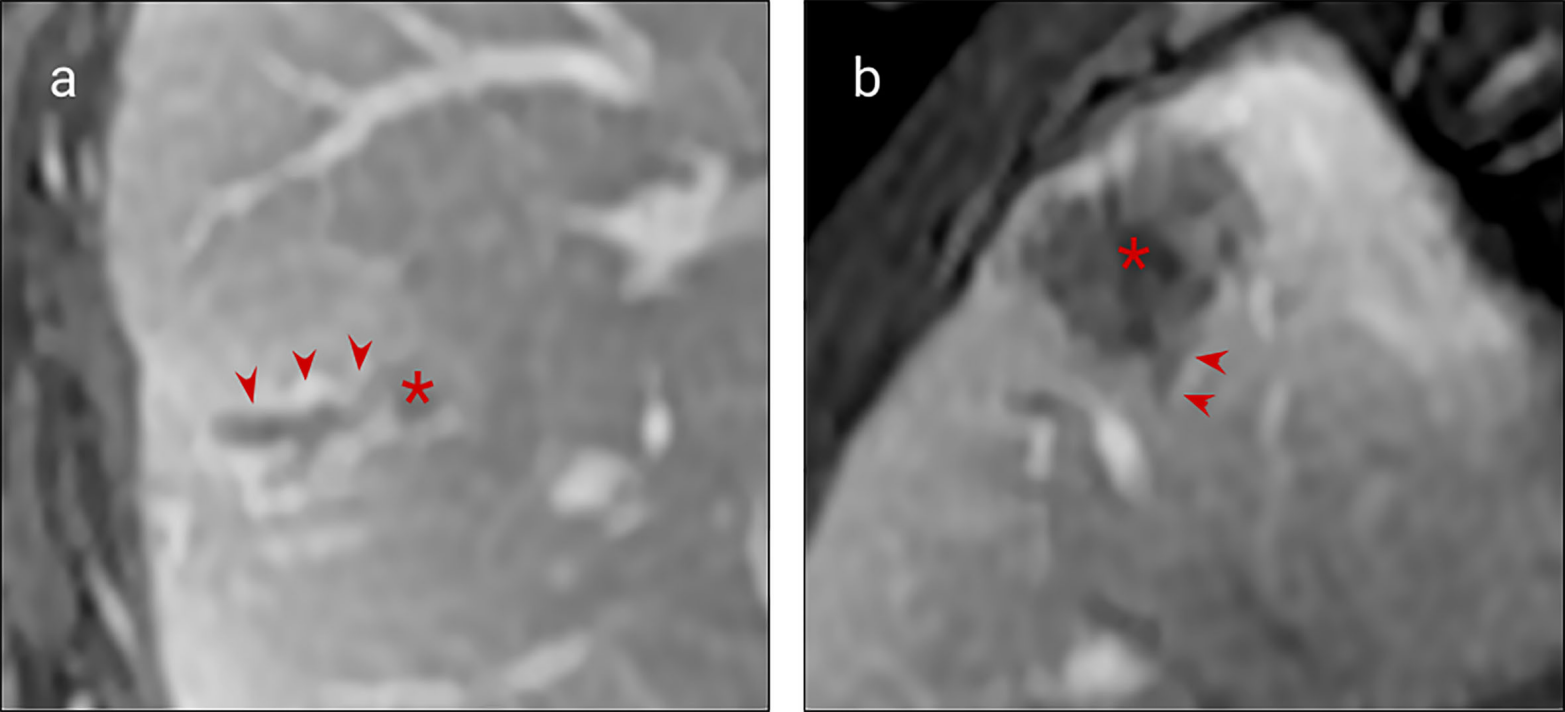
MRI examples of histologically confirmed CRLM with macroscopic vascular invasion. **(A, B)** Portal venous phase gadolinium-enhanced T1 fat saturated MRI shows CRLM lesions (asterisk) with a filling defect within a hepatic vein indicating macroscopic venous invasion (arrows).

Vascular invasion is a common feature of colorectal cancer and its liver metastases. Intrahepatic portal venous invasion local to resected metastases occurs in approximately a quarter of cases (reported range 10-49%) and hepatic venous invasion in approximately one tenth of cases (reported range 5-24%) (10). Like primary colorectal cancer, venous invasion has been associated with poorer clinical outcomes (29). High resolution pelvic MRI has been shown to reliably identify extramural venous invasion of primary rectal cancer (30) and is now a useful prognostic marker. Similarly, although large vessel invasion is less common in CRLM, adjacent venous invasion can be similarly demonstrated (**Figure 2**).

Lymphatic invasion is a less common feature than venous invasion but has again been shown to be associated with poorer outcomes (31–33). Lymphatic vessels are smaller than the resolution of current imaging techniques, and therefore lymphatic invasion has not been reported to be directly accessible by MRI, although the presence of periportal, retroperitoneal or more distant lymphadenopathy may be a surrogate marker for this pathological feature. Neither biliary nor perineural invasion has been associated with adverse clinical outcomes (10) and there are no imaging studies correlating these features with MRI in CRLM.

### Tumour Composition and Markers of Treatment Response

#### Features of Internal Composition

**Figure 3:**
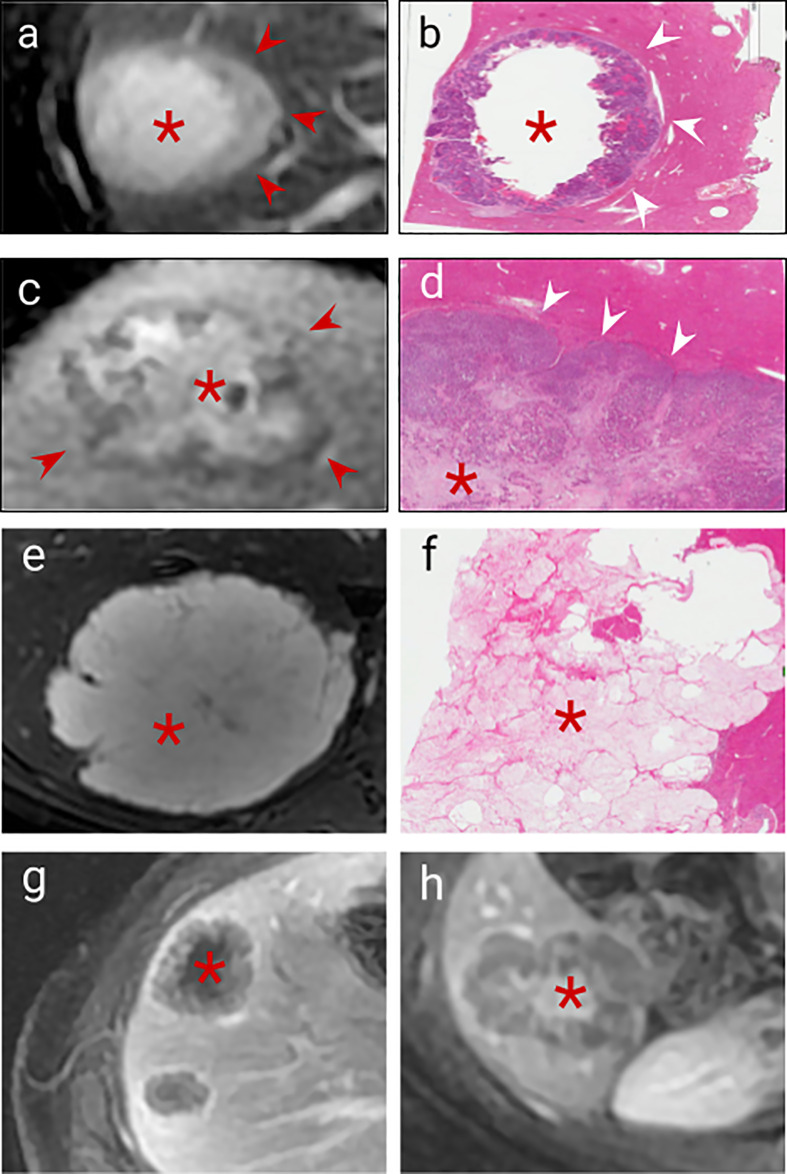
Imaging correlates of internal tumour composition. **(A)**. T2 weighted non-enhanced MRI of a lesion displaying moderately high central T2 signal suggestive of central necrosis (asterisk) and surrounding viable tumour which is higher signal than normal liver (arrows), **(B)**. H&E staining of this CRLM confirms peripheral viable tumour (arrows) with central liquefaction of the metastasis indicating classical Garland necrosis (asterisk), **(C)**. Delayed phase gadolinium-enhanced T1 fat saturated MRI showing avid central delayed enhancement indicating fibrosis (asterisk) with surrounding viable tumour cells which enhance less than normal liver (arrows), **(D)**. H&E staining of this CRLM confirming central fibrosis within the lesion (asteriks) and peripheral viable tumour cells (arrows), **(E)**. T2 weighted sequence demonstrated homogenous high signal mucin (asterisk) **(F)**. H&E staining confirming mucin containing metastasis (asterisk) **(G)**. Delayed phase gadolinium-enhanced T1 fat saturated MRI showing centrally necrotic lesions (no delayed central enhancement, asteriks), **(H)**. Delayed phase Gadolinium enhanced T1 fat saturated MRI showing late central enhancement indicating a centrally fibrotic lesion (asterisk) which contrasts with **(G)** (central necrosis).

The internal composition of tumours differs, comprising variable proportions of tumour cells, fibrosis, necrotic material and, in some cases mucin and calcification. It is also influenced by adjuvant treatment (34, 35). These tumour components have typical MRI features (as shown in **Table 1** and illustrated in **Figure 3**).

**Table 1:** (See corresponding **Figure 3**).

	T2 signal	T1 signal	Enhancement	Diffusion
**Tumour cells** (**Figures 3A–D**)	High signal relative to liver (36)	Low signal relative to liver (36)	Variable, usually reduced enhancement relative to liver (26, 37)	Reduced ADC value (38, 39)
**Necrosis** (**Figures 3A, B, G**)	Variable. (36)	Variable (27, 36)	Delayed enhancement, less than fibrosis (26, 27, 36, 40)	Increased ADC value (41)
**Fibrosis** (**Figures 3C,D,H**)	Low signal relative to liver (27)	Low signal relative to liver (27, 36)	Late enhancement (26, 42, 43)	Increased ADC value (44, 45)
**Mucin** (**Figures 3E, F**)	High signal (46)	Low signal (46)	No enhancement (46)	Increased ADC value (46)
**Calcification**	Signal void (36)	Signal void (36)	None	

Viable tumour cells contrast with normal liver on T1 and T2 weighted sequences (**Table 1** and **Figure 3A**) and are generally hypoenhancing relative to background liver (**Figures 3A, C, G**) (26, 36–38). Necrosis is commonly found in chemo-naïve CRLMs, manifesting as T1 hyperintensity (differentiating it from other components) with variable enhancement, usually less than viable tumour and fibrosis (**Figures 3A, B, G**) (27, 40), with several histological subtypes recognised. CRLM often exhibit classical garland necrosis: areas of necrotic debris, sometimes referred to as ‘dirty’, ‘usual’ or intra-acinar necrosis, surrounded by a rim of viable tumour (**Figures 3A, B**) (27). Fibrosis demonstrates similar T1 and T2 signal characteristics to viable tumour, but typically has delayed gadolinium-enhancement (**Figures 3C, D, H**) (26, 42, 47) which differentiates it from tumour cells. Mucin is identified by pools of homogeneous high T2 and low T1 signal and absent enhancement (**Figures 3** **E, F**). Calcification characteristically presents signal voids on both T1 and T2-weighted sequences (**Table 1**) (36).

Defining the internal composition of metastases may help to categorise tumour biology and thus influence immunological and surgical strategy recently described Consensus Molecular Subtypes (CMS) of colorectal liver metastases (11), can be characterized by particular histopathological features and could be categorized by imaging. The molecular subtyping of liver metastases may be important even in patients when a resected primary tumour specimen is available, as significant discordance exists between primary and metastatic molecular subtypes (46, 48, 49). Within-patient heterogeneity is implicated as the root cause of a variable immunological response between primary and metastatic lesions (50, 51).

Pitroda et al. (11) have proposed three different CRLM subtypes [rather than the four classical colorectal molecular subtypes (52)]. They have identified a stromal metastasis subtype (with epithelial-mesenchymal transition and angiogenesis molecular signatures plus SMAD3 mutation association, subtype 3) which demonstrates significant baseline intratumoral fibrosis (11), in contrast to an immune-subtype (subtype 2) which demonstrates peritumoral lymphocyte infiltration and minimal intratumoural fibrosis (11). As outlined in **Table 1**, fibrosis is readily identified on MRI (27), whereas the degree of angiogenesis/microvascular density could be evaluated by Diffusion Weighted Imaging (DWI) and DCE-MRI enhancement (53–55). These imaging features are amenable to conventional visual, or radiomic derivation, and could provide a CMS prediction, and support personalised treatment. As yet, there have been no studies linking radiological imaging to CMS subtypes, although deep learning technologies have been applied to histological images to predict CMS subtypes and advanced imaging processing techniques (56), which are discussed in more detail below, have been used to classify other molecular features of CRLM.

#### Tumour Viability and Response

Chemotherapy and other systemic treatments, such as immunotherapy, are used in the palliative and neoadjuvant settings. Pre-treatment, proportions of viable and necrotic tumour occurs as a result of intralesional hypoxia and ischaemia as tumour growth outstrips angiogenesis or vascular co-option, and has not been found to be prognostically relevant (34, 57). However, markers suggesting reduction of viable tumour following treatment are useful indicators with a strong prognostic value (34, 57). Here imaging has an important advantage over histopathological assessment of CRLM; the ability to evaluate tumour response before and after treatment. Since the features of chemotherapy response, namely fibrosis and necrosis, are present in chemotherapy-naive metastases, evaluation of response without a baseline assessment becomes more challenging.

**Figure 4:**
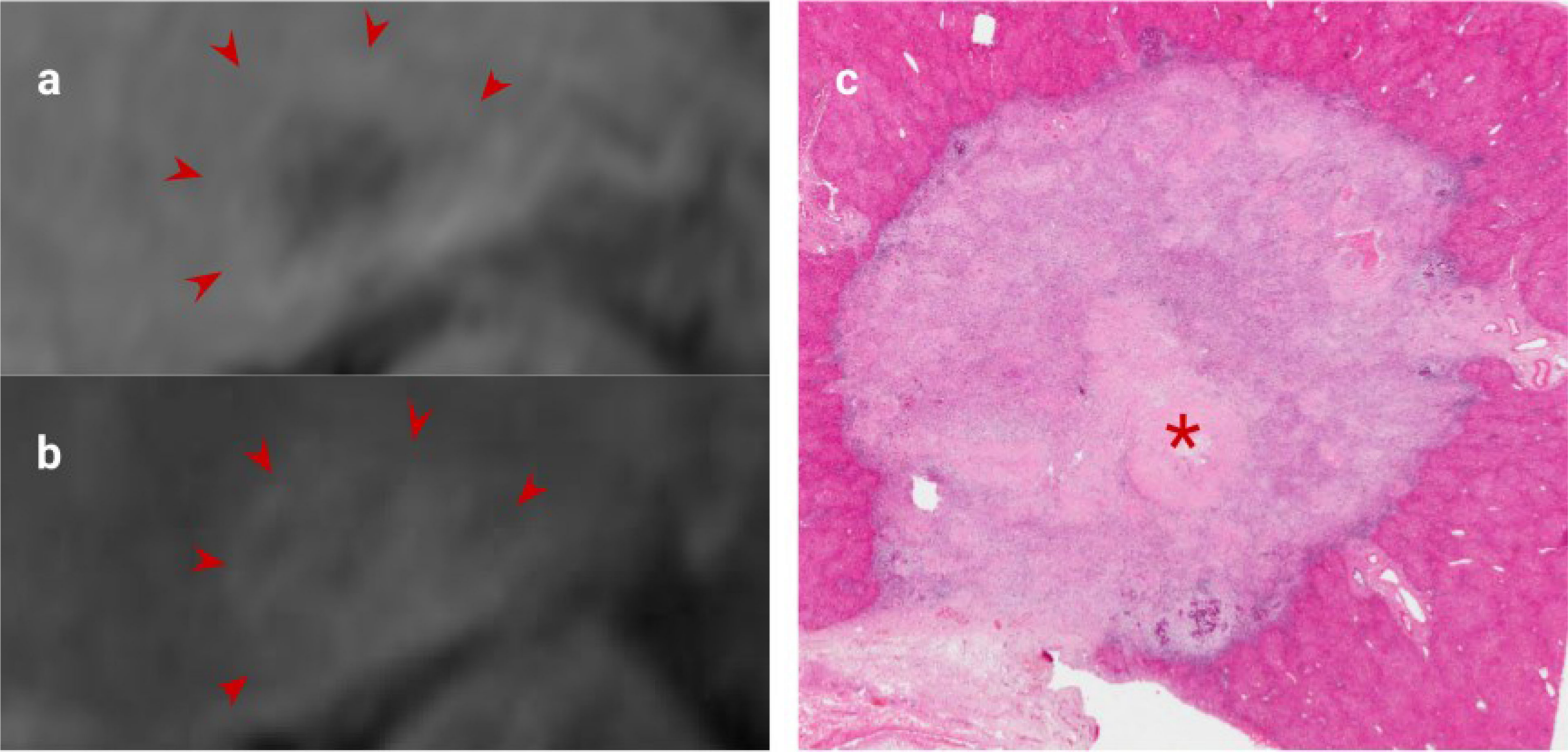
CRLM imaging features following excellent response to Chemotherapy. **(A)**. Portal venous phase gadolinium-enhanced T1 fat saturated MRI showing decreased enhancement relative to liver (arrows), **(B)**. Delayed phase MRI showing increased enhancement relative to liver (arrows), **(C)**. H&E staining of this CRLM confirming almost complete fibrosis of the lesion indicating excellent chemotherapy response. '*' represents the metastasis of interest.

**Figure 5:**
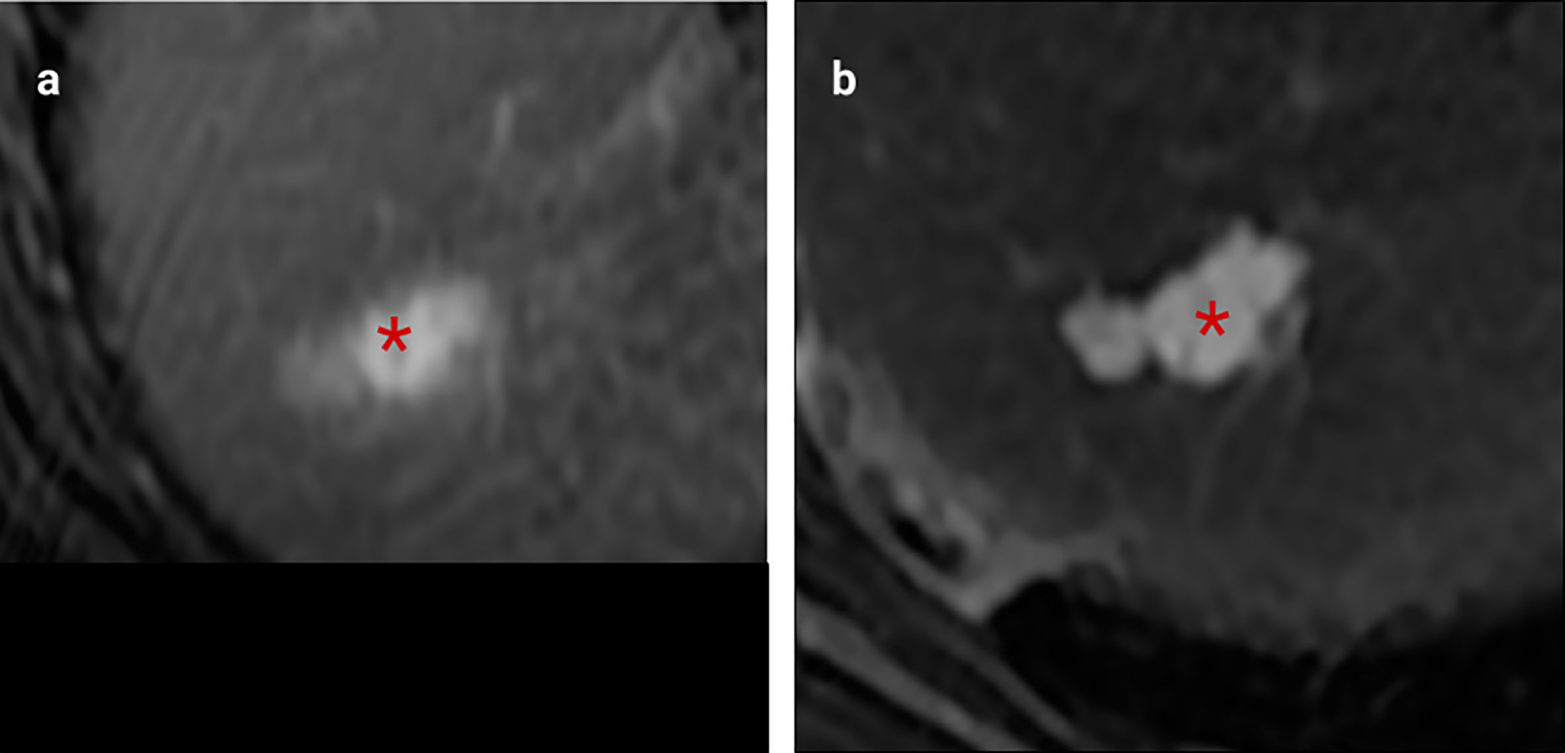
Example of excellent morphological response post-chemotherapy. Both images were obtained using the same MRI scanner performed at 1.5T (GE HDX Twinspeed MR scanner; GE, Milkwaukee, WI) with an 8-channel torso coil. **(A)** T2 weighted MRI showing a poorly defined CRLM with high T2 region of mucin (asterisk). **(B)**. Following neoadjuvant treatment, this lesion demonstrates no change in size but now has a well-defined margin and replacement of intermediate signal cellular tumour with mucin, indicative of a good morphological response (asterisk). There was a complete pathological response at resection.

**Figure 6:**
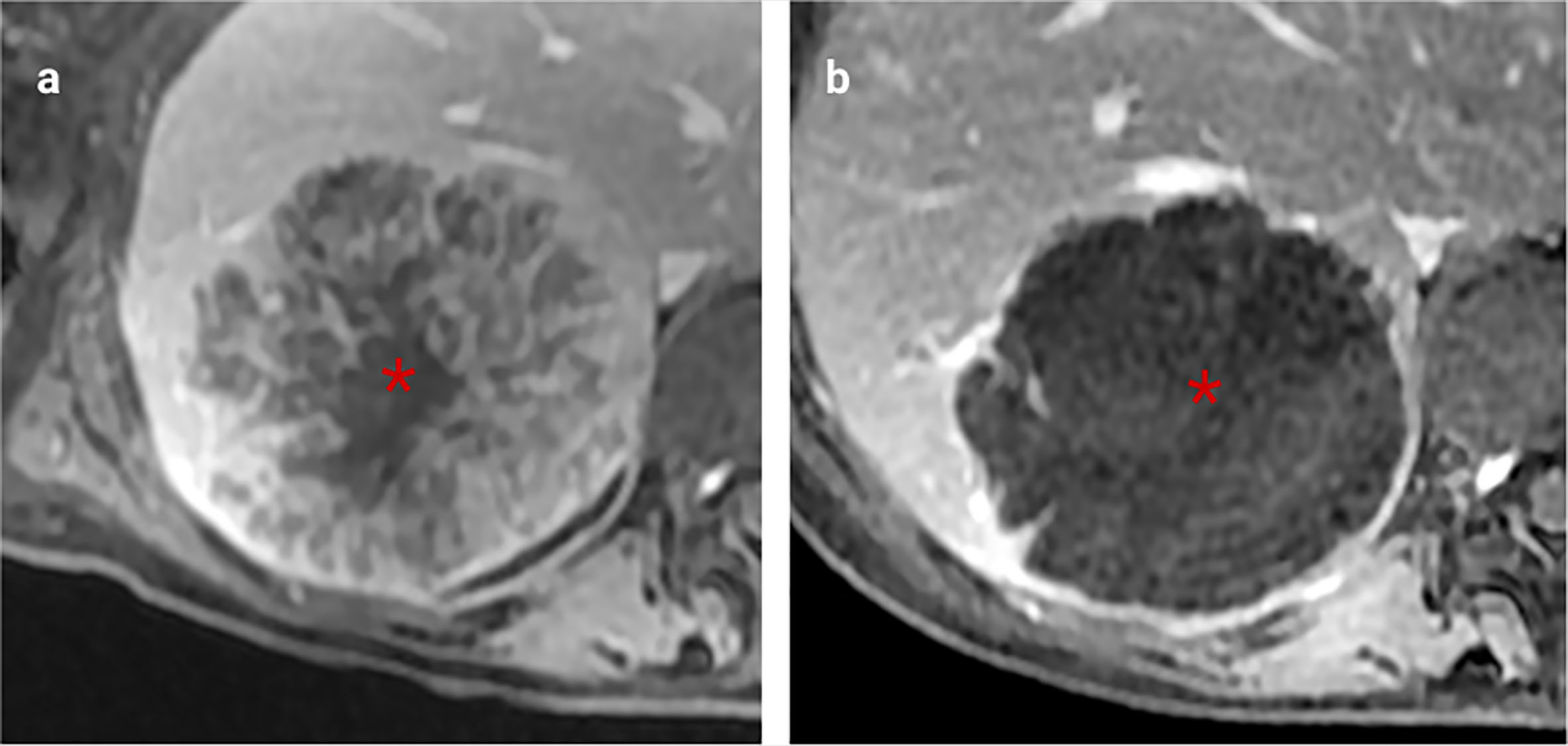
Excellent morphological response to chemotherapy. **(A)**. Heterogenously enhancing CRLM on portal venous phase T1-weighted MRI (asterisk). **(B)**, Post-chemotherapy MRI shows the lesion has become homogenous without a reduction in size, indicating a significant decrease in viable tumour cells.

The method of histological tumour regression grading described by Rubbia-Brandt et al (34) is the most widely used for determining histological response to chemotherapy; it categorises tumour response according to the balance of fibrosis and residual tumour. This method has been adapted from response grading of other tumours such as rectal and oesophageal tumours (58). Imaging response evaluation using the widely adopted RECIST 1.1 criteria, is based on changes in overall lesion size without taking into account changes in tissue composition (59). This is a limitation of the RECIST 1.1 criteria as tumours can demonstrate a reduction in cellularity without a reduction in size (42) (e.g. **Figures 4**–**6**), and some tumours have a low proportion of viable tumour at the outset, limiting the reliability of RECIST assessment (60–62), and its prognostic value. A combined assessment of the viable tumour volume, using both lesion size and tissue characterization may improve treatment assessment, and is already used in tumours such as HCC (63). Ideally automated lesion segmentation, subsegmentation (64) and feature analysis would integrate this process into the imaging pathway.

There is already evidence to support using morphological features to assess response of CRLM to chemotherapy. Lesion fibrosis, demonstrated as late gadolinium enhancement, which is the principle marker of response on pathological assessment, is a feature strongly linked to improved survival outcomes (**Figure 4**) (26, 42, 65). Similarly, CT/MRI morphological changes (illustrated in **Figures 5** and **6**) are better predictors of survival than RECIST [e.g (66–69)]. Adjuncts and alternatives to RECIST, including DWI and FDG-PET response evaluation have been investigated, but are not routinely deployed in clinical practice.

## Towards Comprehensive Imaging Characterization of CRLM: Opportunities and Challenges

The major benefit of imaging is the capacity to perform repeated, *in vivo* assessment of multisite disease, which is a particular advantage in the metastatic setting. In this article, we have described the basis for more complete morphological characterization of CRLM (22). A further strength of imaging is the potential to perform multiparametric imaging, including functional imaging techniques. Functional imaging allows physiological evaluation of tumours, and can aid determination of histological features, and support treatment selection and response. For example, assessment of angiogenesis and microvascular density is possible by diffusion weighted imaging (DWI) (53) and functional vascular assessment by DCE-MRI (54, 55). These techniques could also be used in identifying molecular subtypes of CRLM. Response to antiangiogenic treatment can be monitored with DCE-MRI (54, 70) and this can improve treatment stratification in clinical practice and trials.

Molecular imaging techniques, in particular PET/CT, can provide further functional assessments of CRLM. FDG-PET is well-established in oncological practice, and routinely acquired in staging oligometastatic CRC, principally for detecting disease that would be beyond the scope of local therapy. However, FDG-uptake can also be used as marker of hypoxia, and as a prognostic marker and for response evaluation (71). The potential applications for novel molecular agents to image specific disease features and process are diverse, with hypoxia imaging agents such as 18F-FMISO (72) and antibody-based imaging for CEA (73) are under investigation in the research setting.

However, despite the potential for multiparametric structural, functional and molecular imaging to provide a more comprehensive assessment of CRLM, there are limitations. While these techniques might be valuable, and are often integrated into trials, complex multimodality assessments are challenging in the clinical workflow, and reliably and repeatedly combining information from multiparametric imaging is difficult for human observers. A second major technical challenge to the use of imaging for assessing tissue features of CRLM is the achievable spatial resolution. For MRI, the in-plane spatial resolution is typically in the region of 1mm^2^. Although higher spatial resolutions can be achieved, an important feature of liver imaging is managing respiratory and, to a lesser extent, cardiac motion, which limits acquisition time and spatial resolution achievable in the upper abdomen. This issue is compounded through tissue features which can be substantially smaller than the imaging resolution, which may preclude accurate assessment of some features and place a limit on the achievable performance of imaging.

These two key challenges may be overcome by developments in image acquisition and analysis techniques. MRI acquisition will increasingly use computer-assisted techniques to decrease acquisition time and improve image resolution, for example through the use of ‘super-resolution’ techniques, which may improve the potential for tissue feature assessment by imaging (74). Secondly, improved motion correction and co-registration techniques can help overcome issues with between-acquisition motion (75).

The most promising opportunity within radiology is the incorporation of machine learning in image interpretation. Analysis of tumour features has historically relied on expert radiological assessment of imaging features. However, modern radiomic image analysis can be used to extract high-dimensional data from medical imaging (76), and machine learning techniques can be used for both automated segmentation (77) and feature analysis (78) to produce imaging biomarkers from medical imaging. These analysis strategies can more easily combine multiparametric imaging than a human observer, will remove observer variability, and can become an automated component of the image interpretation pipeline, which would be critical for clinical implementation in patient care.

Several key hurdles must be overcome before radiomics and machine learning becomes robust enough to influence patient care in daily clinical practice. Firstly, many radiomics and machine learning studies on CRLM have been conducted on relatively small datasets (42, 66, 76, 78). Studies conducted on small datasets therefore are at put a Radiomic algorithm at risk of ‘overfitting’ the data (creating an algorithm too specific to a limited pool of data), reducing the generalisability of the study findings (79, 80). The scientific community needs large annotated databases to derive and validate image analysis tools, however the practicality and ethics of sharing scans across multiple institutions, acquired through different and evolving techniques, is an ongoing challenge (79). Aside from acquiring larger, accurately labelled datasets, development of advanced radiomic techniques hold promise for minimising this issue (including various unsupervised clustering methods), but they are unlikely to be the solution without improved sources of data (79, 81, 82).

Even assuming a robust algorithm and analysis platform is developed in the research setting, adapting them into a convenient final product for use in the routine clinical workflow is a further challenge. In addition, many radiomic techniques are time-consuming from an operator perspective with careful lesion contouring required (76, 81). Automated segmentation technologies and other assisted analysis tools will be crucial to ensure workflow ensure clinical adoption.

An important consideration for imaging biomarkers in cancer is the development of alternative techniques for *in vivo* tumour assessment, in particular circulating tumour biomarkers such as circulating tumour DNA (83). However, as these tissues do not allow spatial localization of tumours, it is likely, particularly in the metastatic setting, that imaging can provide complementary information, and the combination of technologies can offer a more comprehensive toolkit to accurately phenotype disease.

## Conclusion

This review has highlighted the potential for advanced imaging to provide *in vivo* characterization of clinically relevant histopathological features of CRLM. We have outlined the imaging findings of these histopathological features, focusing on the tumour-liver interface, intralesional component analysis, and morphological response assessment following systemic or liver-directed treatment. *In vivo* assessment of the tumour-liver interface has the potential to play and important role in defining the surgical approach and chemotherapy selection. As well as improving our characterisation of response to chemotherapy, imaging analysis of internal tumour components could play an increasingly important role as predictors of CRLM molecular subtypes. However, in the absence of studies providing robust validation of imaging techniques to report these features in practice, our assessment of CRLM by imaging is limited to documenting their size, number and location.

Advanced analysis methods, such as radiomics and machine learning, will be crucial tools in developing and validating novel imaging biomarkers for CRLM. However, these rely on curated and annotated datasets of sufficient size to build reliable algorithms, which is likely to require the cross-institutional collaborations that have been achieved in pathology and molecular biology.

## Author Contributions

DM was involved with literature review, and writing and editing the manuscript. MT was involved with literature review, and writing and editing the manuscript. FG was involved with writing and editing the manuscript. DB was involved with writing and editing the manuscript. RG was involved with writing and editing the manuscript. JP was involved with writing and editing the manuscript. AH was involved with writing and editing the manuscript. JF was involved with literature review, writing and editing the manuscript. All authors contributed to the article and approved the submitted version.

## Conflict of Interest

The authors declare that the research was conducted in the absence of any commercial or financial relationships that could be construed as a potential conflict of interest.

## Publisher’s Note

All claims expressed in this article are solely those of the authors and do not necessarily represent those of their affiliated organizations, or those of the publisher, the editors and the reviewers. Any product that may be evaluated in this article, or claim that may be made by its manufacturer, is not guaranteed or endorsed by the publisher.
